# Furuncular Inflammation of the External Auditory Canal

**Published:** 1888-04

**Authors:** S. Latimer Phillips

**Affiliations:** Savannah, Ga.


### FURUNCULAR INFLAMMATION OF EXTERNAL AUDITORY CANAL.

BY S. LATIMER PHILLIPS, M. D., SAVANNAH, GA.

One of the most annoying and painful affections of the ear we meet with in daily practice is a circumscribed inflammation of the cutaneous and subcutaneous tissues of the external auditory canal. This disease is never seen in infants and very young children, but in adults is a comparatively common affection. Buck says it constitutes "not less than two per cent. of all the forms of ear disease which come under the aurists' observation."

As yet our knowledge of the causes of this form of inflammation is not very great. There most always seems to be some fault in the system at large, the general health not being up to par. Those run down by dissipation seem to be more predisposed to this disease than those who take better care of themselves. Young women who, from fashion or other causes, keep late hours, eat irregularly, and then not wholesome food, are liable to have crops of boils in their ears. I had recently a case of that sort under my care. A young lady who was largely engaged in music through the day, her nights being her only leisure time, were spent in entertaining her friends until late; consequently, her health became impaired. She suffered from indisposition and lassitude, and with this came a crop of boils in her ears. She took a trip into the country for her health, which improved, and with it her ears. But as soon as she came back home, and returned to her work and late hours, all her troubles came back on her, boils and all. These she continued to have at varying intervals until she mended her ways.

The entrance of sea-water into the canals, as well as the irritation caused by a continual flow of pus from the tympanum, are said to be not uncommon causes. Chronic eczema of the canal is also a fruitful one; but possibly the picking and rubbing consequent upon the itching may have something to do with it.

From personal observation, I should say this disease was more frequent in females than males, though I have no statistics to prove this. Some aurists hold that irregular menstruation has much to do with this form of inflammation, and at the climacteric period it is likely to occur. Imperfect digestion and malassimilation, however, appear to be the chief underlying cause.

Dr. Lawingberg states that he has found a specific microbe in the blood removed from a recently opened boil in the auditory canal. Upon this is based our antiseptic treatment, which seems to have met with some success.

Oftentimes there appeared to be almost epidemics of this trouble. In fact, they have been reported in Vienna and Paris. The spring appears to be the season at which they are more prone to make their appearance. Cases have been seen in which the patient was a sufferer from diabetes; but as general furunculosis in this disease is not an uncommon symptom, it is not surprising that the auditory canal should at the same time be attacked.

Sometimes there are constitutional symptoms, fever, constipation, etc., but usually the trouble is limited. In the first onset there will be a fullness in the ear, and as the disease advances, throbbing and pain. Tinnitus is not apt to be present, as there is no pressure on the foramen ovale through the membrana tympani and chain of bones. Pain may be severe, extending through side of face and head, or so slight that the patient will barely complain of it, and consult the physician only on account of the unpleasant sensation of fullness in the ear. Dullness of hearing is a prominent symptom; it varies from a little hardness of hearing to an almost absolute deafness when the swelling is sufficiently large to fill up the canal and thus obstruct the course of the sound waves. When left to itself the pain lasts from three to eight days, when the abscess ruptures; this is dependent upon the depth of the focus of inflammation. After the rupture, pain usually ceases, and there is a discharge from the canal for some-time; but in some cases pain may remain even after the furuncle is thoroughly opened. The seat of the abscess is generally in the cartilaginous portion of the canal, though some authors speak of it being found in the bony part. When there, it is apt to be intimately connected with the tympanum and tympanic membrane, and so much so that it could hardly be called an independent affection of the external auditory canal.

Very little trouble is experienced in the diagnosis. In taking hold of the pinna and moving it about, intense pain is experienced, which would not be felt if the inflammation was confined to the tympanum, on account of the immobility of that part. On inspection with the reflected light, some point of elevation or redness will be seen. If not, a probe can be gently passed about in the canal and the most sensitive point made out. When the inflammation has been severe, and there is much swelling, it will be quite hard to introduce a speculum, and impossible to get a view of the drum membrane.

If there are no complications, the prognosis is always good, under proper treatment, pain disappearing, and after all subsidence of inflammation, hearing being fully restored.

In the treatment the causes should be diligently sought for and removed. Digestion must be looked after, and if at fault put to rights. When the patient is feeble, suffering from want of appetite, lassitude, with a feeling of weariness and disposition to do nothing, I find the concentrated extract of malt to be valuable; it gives tone to the system, and under its influence the patient soon brightens up. It improves the appetite; digestion and assimilation are better, and with these comes an improvement in the local condition. Many of the cases require some one of the ferruginous preparations, and at times bark is needed; at other times small doses of nux vomica become useful; plenty of out-door exercise is necessary, and when practicable horseback rides should be indulged in. Of course all forms of dissipation must be given up by those who are in the habit of indulging in them. Many a young lady who suffers from this form of ear trouble only gets well when the gay winter season is over and she gets the much needed rest.

Leeches are sometimes advised to be applied about the ear in the local treatment of this disease. They do but little, if any, good, and had better not be used. In inflammation of the deep parts of the ear they are of great value, but in this superficial form it is not so. Warm water instilled in the canal will, in a measure, give relief from pain and cause the abscess to point sooner. Poultices would be of as much value here as in other abscesses, but from the anatomy of the part are almost useless. In my opinion, a free and deep incision in the furuncle, as soon as diagnosis is made, is by far the best method to pursue. A free flow of blood from the part, which we get after cutting it, lessens the congestion there, and thus has a tendency to modify the intensity of other furuncles, if not entirely to prevent their formation. In doing this operation the head of patient should be left free, so if he should jump it will be away from the knife and no harm will be done. This operation can most always be done without an anæsthetic. Sometimes, from the nervousness and timidity of the patient, one will have to be given, and ethyl bromide will be the best. With it the patient can be put to sleep and the operation done in a few minutes. Coming to, he can get up and go immediately out without any of the annoyances which generally follow chloroform or ether narcosis. The operator should sit facing a bright light, and by means of the focus mirror illumine well the canal. The knife usually employed for these operations is a small curved bistoury. The cut is made deep, downwards and outwards. Prof. Gruber has devised a tenotome which is very valuable when the furuncle is deep in the canal, or the membrana tympani is to be perforated. It consists of a handle in which a number of small knives of varying kinds may be fitted and firmly held in position. It is bent at an angle so that the operator's hand will not interfere with his light. When pus has formed and the opening made, it may be squeezed out of the cavity with a delicate curette. It would be well to wash out the canal with a warm solution of bichloride of mercury (1 to 20,000) so that any bacilli that should be present in the blood or pus may be destroyed. Vaseline can be smeared over the parts to keep them soft and will lessen the induration. I think sometimes, in the early stages, and when the point of inflammation was small, I have seen resolution take place after a free application of the following ointment:

| | |
|---|---|
| Hydrg. Ox. Flav. . . . . . . . . . . . | gr. ii. |
| Vaseline . . . . . . . . . . . . . . . | ʒi. |

Some aurists (Sexton and Burnett) claim that sulphide of calcium, in small doses internally, has a tendency to prevent formation of auditory furuncles. I have used it, in one-tenth of a grain doses, three times a day, but saw no appreciable effect. After an operation, a small piece of wool may be placed in the canal to protect it for a while.

---

THE *Gazette des Hôpitaux*, of January 21st, publishes a report presented by M. Troisier to the Société Médicale des Hôpitaux, at the meeting of January 13th, on M. André Petit's observations on the existence of enlarged supra-clavicular glands in cases of cancer of the uterus. M. Troisier stated that he had met with several cases of uterine and ovarian cancer, accompanied by enlarged glands, in the supra-clavicular region in which no intermediary glands could be detected elsewhere. This symptom may also be met with in cancer of the abdomen as well as in cancer of the stomach or of the thorax. It should be looked upon as contra-indicating surgical interference in such cases.—*British Medical Journal.*

